# Quantification of Circulating Endothelial Progenitor Cells Using the Modified ISHAGE Protocol

**DOI:** 10.1371/journal.pone.0013790

**Published:** 2010-11-03

**Authors:** Caroline Schmidt-Lucke, Stephan Fichtlscherer, Alexandra Aicher, Carsten Tschöpe, Heinz-Peter Schultheiss, Andreas M. Zeiher, Stefanie Dimmeler

**Affiliations:** 1 Department of Molecular Cardiology, Internal Medicine III, J.W. Goethe University, Frankfurt, Germany; 2 Department of Cardiology and Pulmonology, Charité, Berlin, Germany; Brigham and Women's Hospital, United States of America

## Abstract

**Aims:**

Circulating endothelial progenitor cells (EPC), involved in endothelial regeneration, neovascularisation, and determination of prognosis in cardiovascular disease can be characterised with functional assays or using immunofluorescence and flow cytometry. Combinations of markers, including CD34+KDR+ or CD133+KDR+, are used. This approach, however may not consider all characteristics of EPC. The lack of a standardised protocol with regards to reagents and gating strategies may account for the widespread inter-laboratory variations in quantification of EPC. We, therefore developed a novel protocol adapted from the standardised so-called ISHAGE protocol for enumeration of haematopoietic stem cells to enable comparison of clinical and laboratory data.

**Methods and Results:**

In 25 control subjects, 65 patients with coronary artery disease (CAD; 40 stable CAD, 25 acute coronary syndrome/acute myocardial infarction (ACS)), EPC were quantified using the following approach: Whole blood was incubated with CD45, KDR, and CD34. The ISHAGE sequential strategy was used, and finally, CD45^dim^CD34^+^ cells were quantified for KDR. A minimum of 100 CD34^+^ events were collected. For comparison, CD45^+^CD34^+^ and CD45^−^CD34^+^ were analysed simultaneously. The number of CD45^dim^CD34^+^KDR^+^ cells only were significantly higher in healthy controls compared to patients with CAD or ACS (p = 0.005 each, p<0.001 for trend). An inverse correlation of CD45^dim^CD34^+^KDR^+^ with disease activity (r = −0.475, p<0.001) was confirmed. Only CD45^dim^CD34^+^KDR^+^ correlated inversely with the number of diseased coronaries (r = −0.344; p<0.005). In a second study, a 4-week de-novo treatment of atorvastatin in stable CAD evoked an increase only of CD45^dim^CD34^+^KDR^+^ EPC (p<0.05). CD45^+^CD34^+^KDR^+^ and CD45^−^CD34^+^KDR^+^ were indifferent between the three groups.

**Conclusion:**

Our newly established protocol adopted from the standardised ISHAGE protocol achieved higher accuracy in EPC enumeration confirming previous findings with respect to the correlation of EPC with disease activity and the increase of EPC during statin therapy. The data of this study show the CD45^dim^ fraction to harbour EPC.

## Introduction

In 1997, Asahara et al. [1] reported the isolation of putative endothelial progenitor cells (EPC) from human peripheral blood on the basis of cell surface expression of haematopoietic stem cell (CD34) or kinase insert domain receptor (KDR) markers. They observed that these cells differentiate into endothelial cells *in vitro* and *in vivo*.

Circulating EPC correlate with risk factors for coronary artery disease [2], participate in endothelial regeneration [3], and can be characterised with functional assays [2], [4] or immunofluorometric methods such as flow cytometry. We and others have shown that EPC independently predict future cardiovascular events in patients at risk [5], [6]. The use of defined culturing assays is handicapped by difficulties in standardisation and the prolonged assay time. Several combinations of markers have been employed by us and other groups to assess circulating EPC [7], [8]. Currently, the combinations of CD133^+^CD34^+^KDR^+^, CD34^+^KDR^+^, or CD14^+^CD34^low^ [9], [10], [11], are widely used to define or select cells that express properties attributed to EPC.

However, the lack of an established and generally accepted marker combination and of a standardised protocol with regards to reagents and gating strategies may account for the widespread interlaboratory variations in quantification of EPC [12]. This hampers comparison and cooperations between different laboratories.

We, therefore, developed a protocol adapted from the standardised so-called ISHAGE (International Society of Hematotherapy and Graft Engineering) [13] protocol for enumeration of haematopoetic stem cells to enable comparison of clinical and laboratory data. Since recent studies suggest that particularly the fraction of CD45^−^ cells may harbour the “true” circulating EPCs [14], we included the pan-leukocyte marker CD45 in the analysis. Quantification was performed strictly following the sequential gating strategy [13] and CD34^+^ cells were additionally subdivided in CD45^−^, CD45^ldim^ and CD45^bright^ subpopulations.

## Materials and Methods

### Patients and control subjects

The study population comprised 27 control subjects and 70 patients with coronary artery disease recruited in two centres. Twentyseven healthy subjects without any evidence of CAD by history and physical examination served as a control group. Forty patients had stable coronary artery disease (CAD), defined as angiographically documented CAD and the absence of acute coronary syndromes (ACS) for 3 months before blood samples were drawn. Thirty patients were studied with unstable CAD defined as de novo angina, crescendo angina, or angina at rest. Patients with ACS were further stratified for troponin T positivity to account for potential effects of myocardial necrosis on EPC levels. Time to first and time to the last onset of symptoms, repetitive angina, and as well as duration of symptoms were assessed. Extent of disease was quantified in all patients by the number of coronary arteries affected. Inclusion criteria were age from 18 to 85 years, signed written informed consent, documented CAD for patients with stable CAD or ACS (Braunwald IIIB) or acute myocardial infarction (both non–ST-segment and ST-segment myocardial infarction). Exclusion criteria were clinical or biochemical evidence for the presence of concomitant inflammatory disease, chronic renal insufficiency (serum creatinine <1.4 mmol/L), impaired left ventricular ejection fraction (<45%), autoimmune or malignant disease, thrombocytopaenia (<100 000/L), anaemia (haemoglobin <8.5 g/dL), inability to understand the consent form, participation in or consent to participate in another study, previous coronary bypass surgery, severe peripheral arterial occlusive disease, or atrial fibrillation.

A subgroup of ten patients with angiographically documented stable angina, who had not previously taken statins were included into a second study. Here, all patients received a de-novo therapy of 40 mg atorvastatin per day. Exclusion criteria were previous treatment with a statin, acute coronary syndromes in the last 4 weeks, or any of the above mentioned exclusion criteria.

All study participants gave written informed consent, and the study was approved by the ethics committee of J.W.Goethe University (Frankfurt, Germany).

### Definition of Risk Factors for Coronary Artery Disease

Risk factors were defined as follows: Hypertension was defined as a history of hypertension for >1 year requiring the initiation of antihypertensive therapy by the primary physician, or a repetitive measurement of hypertensive resting blood pressure. Smoking was defined as a history of smoking for >2 pack-years and/or smoking in the last year. Positive family history for coronary artery disease was defined as documented evidence of premature coronary artery disease in a close relative (men <55 and women <65 years of age) [15]. Diabetes mellitus was defined as the need for oral antidiabetic drug therapy or insulin use. We calculated a score giving the risk of an individual subject to develop cardiovascular disease by considering age >65 years, male sex, hypertension, diabetes, smoking, and positive family history for coronary artery disease as single cardiovascular risk factors [16].

### Flow cytometry quantification of EPC, following the ISHAGE (International Society for Hematotherapy and Graft Engineering) gating strategy

**Figure 1:**
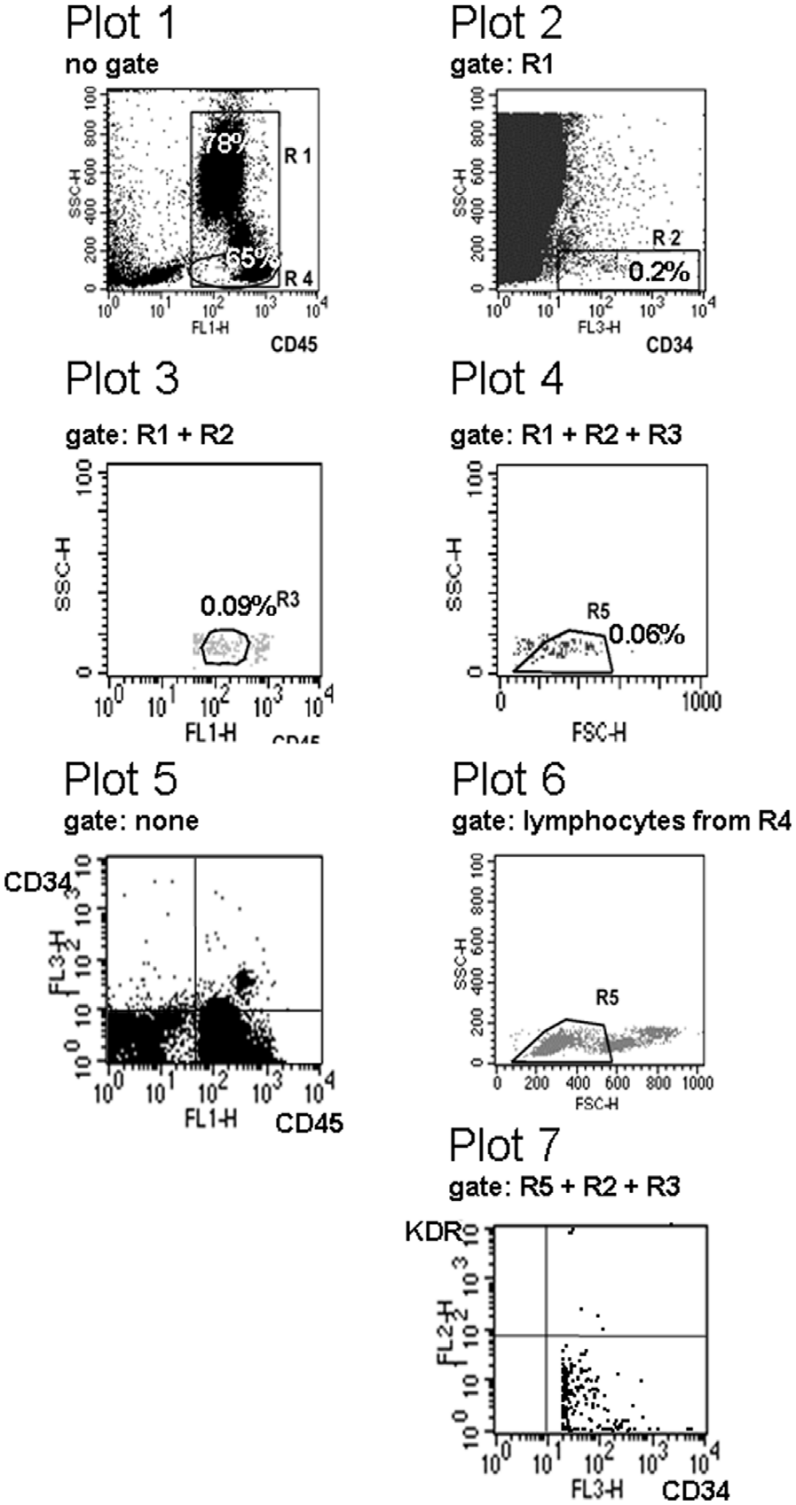
Flow cytometry quantification of EPC following the ISHAGE (International Society for Hematotherapy and Graft Engineering) gating strategy (see Methods section).

EPC were quantified using the following approach: EDTA collection tubes were transported on wet ice into the laboratory and proceeded within 2 to 3 hours. FcR-blocking was added and incubated for 10 minutes. All staining procedures were performed on ice. 1 ml of whole blood was incubated with 4 µl of CD45 (FITC; Beckton Dickinson), 8 µl of KDR (PE; R&D Systems, cat no. FAB 3578), and 4 µl of CD34 (PerCP; Beckton Dickinson). The samples were lysed before flow cytometry analysis. The ISHAGE sequential strategy [17] was used (see Figure 1).

An initial gate (R1 plot 1) is set on a CD45 vs. side scatter (SSC) dot plot to contain all CD45^+^ events including CD45^dim^ and CD45^bright^. This will exclude CD45^−^ events (i.e. red blood cells, platelets and other debris). The lower limit of CD45 expression is adapted from a CD34 vs. CD45 histogram performed on ungated data (plot 5). R1 also contains a gate R4, which defines lymphocytes as CD45^bright^SSC^low^ cells.

The events in gate R1 are then displayed on a CD34 vs. SSC dot plot (plot 2) and a second gate (R2) is produced to include the cluster of CD34^+^ events. The third plot (plot 3) is obtained by plotting the events that fulfil the criteria of gates R1 and R2 (i.e. sequential gating). Cells forming a cluster of blasts with characteristic low SSC and low CD45 fluorescence (SSC^low^CD45^dim^ cells) are then gated on this third plot to produce a third region (R3) (plot 3). To differentiate between CD45^dim^ and CD45^bright^ cells, the right margin of the CD34^+^ cells in plot 5 serves as cut-off. At this step, the gating strategy has defined CD34^+^CD45^dim^ cells. In case, CD45^bright^ cells were investigated for comparison, gate R3 was shifted to the right population visible in plot 3. Finally, the events fulfilling the criteria of all three gates (R1, R2 and R3) are then displayed on a forward light scatter (FSC) vs. SSC dot plot to confirm that the selected blasts fall into the lymphocyte region (R5) (plot 4). The lymphocyte region (plot 6) was adjusted from a SSC vs. FSC plot gated on lymphocytes from R4 (plot 1), employing only small lymphocytes (FSC^low^, R5 in plot 6).

CD45^dim^CD34^+^KDR^+^ endothelial progenitor cells are then deducted from the upper right quadrant of plot 7, defining CD34^+^KDR^+^ cells, when falling into all three regions (R2, R3 and R5).

Isotype controls are not required because the gating strategy excludes cells that non-specifically bind anti-CD34. CD45^dim^CD34^+^KDR^+^ cell determinations are performed in duplicate and the mean CD45^dim^CD34^+^KDR^+^ value is used. Similarly CD45^−^CD34^+^, or CD45^bright^CD34^+^ cells were quantified for KDR expression.

Around 250,000 events in the gate R1 were acquired (approximately 290,000 total events), and a minimum of 100 CD34^+^ events were collected, according to recommendations. Intraindividual correlation was r = 0.92 (p<0.001) and inter-class correlation was r = 0.93 (p<0.001) for CD45^dim^CD34^+^KDR^+^, and thus comparable to our previously published data (r = 0.86, p<0.001) [5].

### Statistical analysis

Continuous variables were tested for normal distribution with the Kolmogorov-Smirnov test. Comparisons between the two groups were analysed by t test (two-sided) for normally distributed variables or ANOVA for normally distributed variables with more than two subgroups, and by the Kruskal-Wallis test for non-normally distributed variables. Post hoc range tests and pair-wise multiple comparisons were performed with the t test (two-sided) with LSD adjustment. Not normally distributed continuous variables (CD45^dim^KDR^+^CD34^+^ EPC, HDL cholesterol) were compared by the Mann-Whitney-U test. Comparison of categorical variables was generated by the Pearson χ^2^ test. Data are expressed as mean ± SD, unless otherwise stated. Multivariate linear regression analysis and nonparametric bivariate correlation (Spearman rank correlation coefficient) were used to correlate circulating EPC counts with cardiovascular risk factors. To identify independent determinants of EPC numbers, a multivariate linear regression analysis for various cardiovascular risk factors was performed. Statistical significance was assumed, if a null hypothesis could be rejected at p≤0.05. All statistical analysis was performed with SPSS 16 (SPSS Inc.).

## Results

### Patient Characteristics

The baseline characteristics of the 97 subjects are summarised in Table 1. As expected, patients with documented coronary artery disease had a significantly higher number of risk factors, were significantly older, and more frequently were treated with statins, platelet inhibitors, and ACE inhibitors/AT-1 receptor blockers. Due to the intensive statin treatment in patients with CAD, LDL-cholesterol was significantly lower compared to controls. In addition, patients with ACS were less likely to be treated with statins, antithrombotics, and ACE inhibitors/AT-1 receptor blockers compared to patients with stable CAD prior to inclusion into the study.

**Table 1:** Patients baseline characteristics.

	Control	Stable CAD	ACS	P
N	27	40	30	
Age (years)	53±14	61±11	61±13	Co vs CAD and ACS: <0.05
Male gender	18 (67%)	40 (100%)	25 (83%)	Co vs CAD: p<0.05
CVRF score	1.5±1.2	3.5±1.2	3.0±1.1	Co vs CAD and ACS: <0.001
Hypertension	8 (14%)	31 (77%)	19 (63%)	Co vs CAD and ACS: <0.05
Smoking	4 (15%)	12 (30%)	13 (43%)	n.s.
Diabetes mellitus	1 (4%)	11 (27%)	8 (27%)	Co vs CAD and ACS: <0.05
Family history	5 (19%)	13 (33%)	4 (13%)	n.s.
Troponin T positive	n.a	n. a.	9 (30%)	n. a.
Total cholesterol	194±45	182±40	195±40	n.s.
LDL cholesterol	90±40	49±14	75±40	Co vs CAD: <0.05
HDL cholesterol	82±40	102±37	89±50	
Extent of disease(1-/2-/3 vessel disease)	0	1.9±0.917/10/13	2.0±0.911/9/10	Co vs CAD and ACS: <0.0001
Statin therapy	4 (15%)	24 (60%)	7 (24%)	CAD vs. Co and ACS: <0.05
Aspirin/Clopidogrel	2 (7%)	35 (88%)	11 (38%)	CAD vs. Co and ACS: <0.001
ACE-Inhibitor/AT1-Blocker	5 (19%)	34 (85%)	14 (48%)	CAD vs. Co and ACS: <0.005

CVRF: cardiovascular risk factor score; Patients baseline characteristics for patients with ACS with troponin positive or negative values are similar.

The subgroup of ten patients with stable CAD included in the second study, had a similar cardiovascular risk profile, age, number of diseased coronaries, and cardiovascular drugs, with the exception of statins, compared to the total group of stable CAD.

### Circulating endothelial progenitor cells

**Figure 2:**
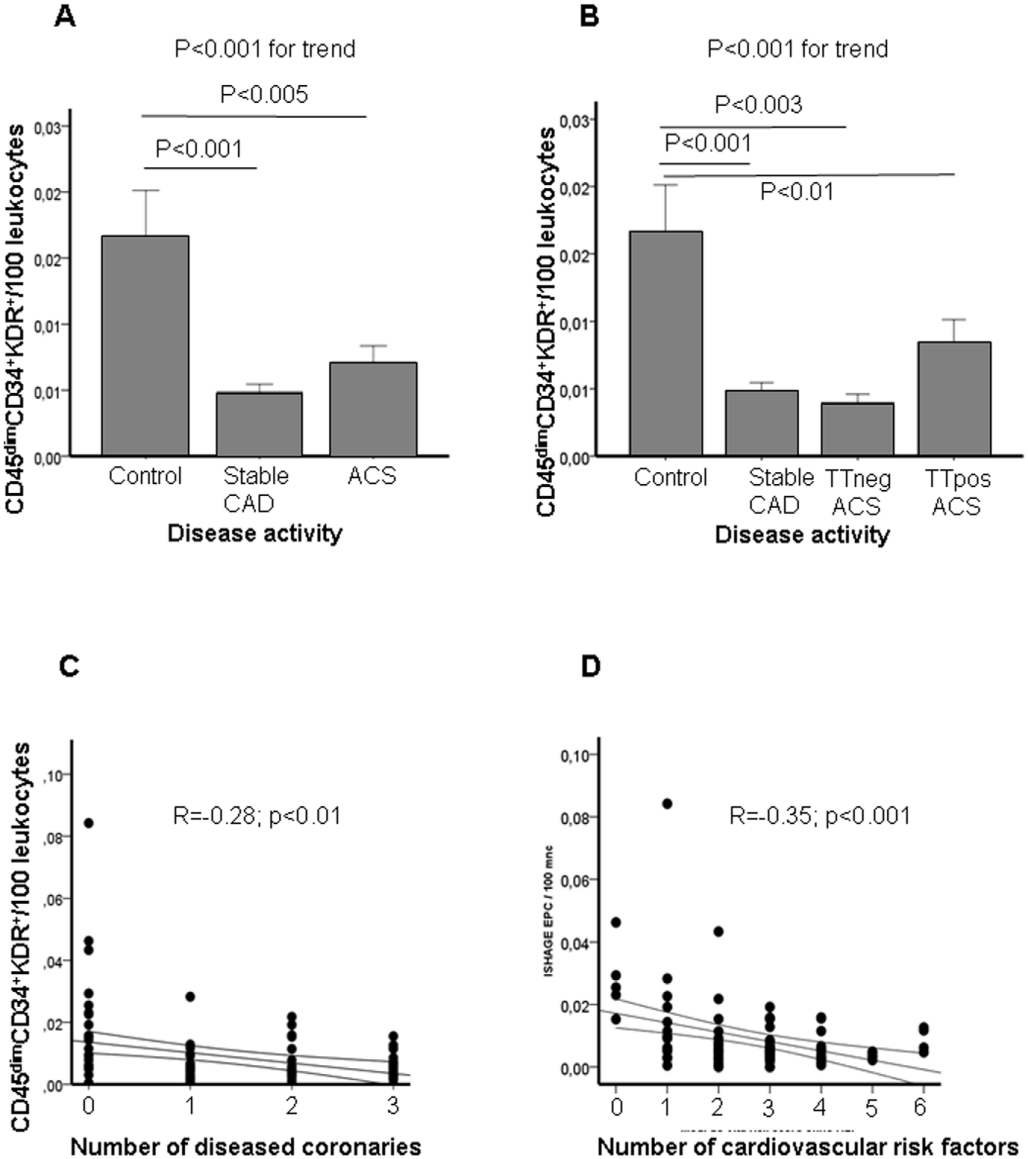
Percentage of circulating CD45^dim^CD34^+^KDR^+^ CPC/leukocytes. (A) in healthy control subjects and patients with stable CAD and ACS. Bars represent mean with SE. (B) in healthy control subjects and patients with stable CAD, troponin T negative (TTneg) ACS and troponin positive (TTpos) ACS. Significance levels on bars represent differences between groups; Significance level for the whole analysis is given above. (C) Correlation of CD45^dim^CD34^+^KDR^+^ CPC/leucocytes with the number of diseased (stenosis>50%) coronaries in healthy control subjects and patients with stable CAD and ACS. (D) Correlation of CD45^dim^CD34^+^KDR^+^ CPC/leucocytes with the number of individual cardiovascular risk factors in healthy control subjects and patients with stable CAD and ACS.

There were significantly higher numbers of CD45^dim^CD34^+^KDR^+^ in controls compared to CAD (0.0167±0.0178% vs. 0.0048± [18] 0.0037%/leukocytes, p<0.001) or ACS (0.0071%±0.0067%/leukocytes, p<0.005; with p<0.001 for trend) (Figure 2A). In contrast to our previous findings [5], patients with ACS did not have lower numbers of circulating progenitors. To assess potential confounders due to ischaemia-induced mobilisation, we subdivided the group of ACS according to the presence of troponin T. Indeed, patients with cardiac necrosis had a strong tendency towards increased numbers of CD45^dim^CD34^+^KDR^+^-cells (p<0.08; Figure 2B). Among patients with ACS, there was an evident tendency (r = 0.37, p<0.07) towards increased number of CD45^dim^CD34^+^KDR^+^-cells with time of first angina, but not with duration of pain or time to last angina. Moreover, CD45^dim^CD34^+^KDR^+^-cells correlated inversely with the number of diseased coronary arteries (r = −0.28; p<0.01; Figure 2C) and with the number of individual cardiovascular risk factors (r = −0.35, p<0.001; Figure 2D). On multivariate analysis, including LVEF (r = −0.28, p<0.01), number of diseased coronaries, disease activity and cardiovascular risk factors, number of cardiovascular risk factor remained the only significant determinant for number of CD45^dim^CD34^+^KDR^+^-cells (t = −2.44, p<0.05, p<0.001 for trend).

On the other hand, CD45^bright^CD34^+^KDR^+^ (control: 0.0097±0.008%, CAD: 0.006±0.006%, ACS: 0.006±0.004%), or CD45^neg^CD34^+^KDR^+^ (control: 0.005±0.003%, CAD: 0.008±0.008%, ACS: 0.007±0.0068%) did not differ between controls and patients with CAD or ACS, nor was there any correlation with diseased coronaries (data not shown).

### Effects of treatment with atorvastatin on levels of circulating endothelial cells

**Figure 3:**
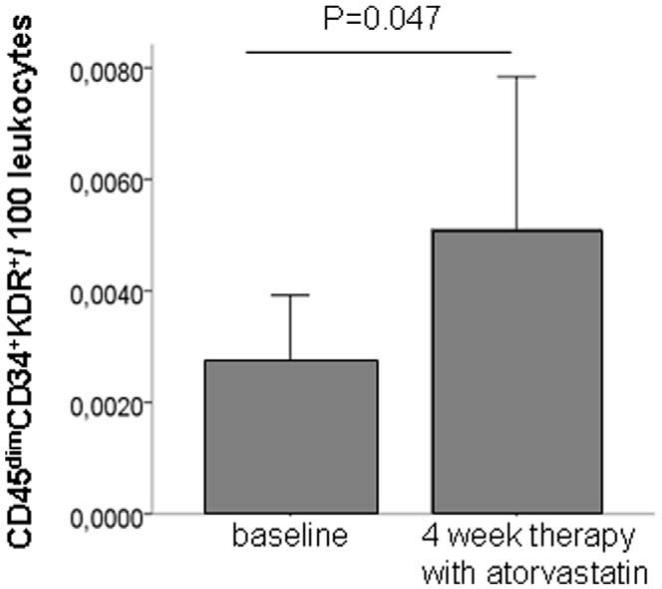
Responses of circulating endothelial progenitor cells (CD45^dim^CD34^+^KDR^+^/leukocytes) in patients treated with de novo 40 mg atorvastatin/day. The left bar indicates the number of progenitor cells before statin therapy, the right bar after 4 weeks of treatment.

In order to assess a potential treatment effect on EPC numbers, 10 patients with stable CAD were treated de-novo with 40 mg of atorvastatin for 4 weeks. As illustrated in Figure 3, statin treatment significantly increased numbers of CD45^dim^CD34^+^KDR^+^ 2.6-fold (p = 0.047), whereas CD45^−^CD34^+^KDR^+^ and CD45^bright^CD34^+^KDR^+^ cells did not change (data not shown).

## Discussion

Undoubtedly, there is a large body of evidence for the regenerative potential of endothelial progenitor cells since their discovery in 1997 [18], [19], [20]. Since then, it has been uniformly shown that numbers of circulating EPC in humans show an inverse association with atherosclerosis [20] and cardiovascular risk [21] and ultimately, independently predict cardiovascular disease progression [5], [6]. Commonly, surface marker combinations include markers for HSC such as lineage depletion and expression of CD34, CD133, and the endothelial marker KDR (VEGFR-2). For quantification of circulating EPC in humans, flow cytometry is widely used.

Nevertheless, there are ongoing debates about the definition of ‘true’ EPC, as well as the availability of a reliable method to assess their quantity and quality, functional status, and therapeutic application. There is a sound body of evidence that bone marrow-derived circulating progenitor cells, presumably of haematopoietic origin without the ability of differentiating into mature endothelium have an important role in vascular homoeostasis. These cells are found in cell fractions staining positive for CD34 and CD34^+^KDR^+^ Generally, to assess CPC function and numbers in humans, there are numerous protocols in different laboratories for culture assays and immunostaining with surface markers. So far, no consensus for a standardised protocol has been reached amongst laboratories that would allow interlaboratory comparability and reproducibility. The use of defined culturing assays is handicapped by difficulties in standardisation and the prolonged assay time. Definition of CPC with angiogenetic potential by surface markers is challenging, given the change in surface marker profiles during the process of mobilisation and maturation. Consequently, most groups use combination of surface markers. But even then, gating strategies are not standardised. Furthermore, the variability associated with enumeration of low-frequency cells (i.e., as low as 0.1% or 5 cells/µl) is exceedingly large [22].

About a decade ago, the need for a rapid and reliable marker for the engraftment potential of haematopoietic stem and progenitor cell (HSC) transplants has led to the development of flow cytometric assays to quantitate such cells on the basis of their expression of CD34 [13]. The standardisation was based on the use of state-of-the-art bright fluorochrome conjugates and the combination of the HSC marker CD34 with CD45 counterstaining. Moreover, gating strategies to separate the CD34^+^ HSC from irrelevant cell populations, as well as inclusion of CD34^dim^ and CD34^bright^ populations were established. Omission of the negative control staining was introduced to facilitate routine flow cytometric measurements. Lastly, it has been agreed to enumerate at least 100 CD34^+^ cells to ensure a 10% precision [22]. During the last decade, the ISHAGE (International Society of Hematotherapy and Graft Engineering) method to detect CD34 HSC in haematological studies has been successfully implemented in multicenter trials [23].

We, therefore, developed a new protocol adapted from the standardised ISHAGE) [13] protocol for enumeration of haematopoietic stem cells to enable comparison of clinical and laboratory data. The sequential strategy for quantification was strictly followed. We added the PE-labelled surface marker KDR to the original protocol. After identification of HSC, immunofluorescence of these cells for KDR was assessed.

Using this protocol, we achieved a high intra- and interclass accuracy. The standardisation of the protocol allowed differently experienced investigators to reproducibly analyse the measurements.

The data obtained with this protocol showed only numbers of CD45^dim^CD34^+^KDR^+^ cells to be significantly higher in healthy controls compared to patients with CAD or ACS. CD45^dim^CD34^+^KDR^+^ correlated inversely with the number of cardiovascular risk factors, even after correction for disease activity and number of diseased coronaries. Apart from the finding of an increase of CPC in patients with myocardial infarction, these findings are well in line with our previously published data [5]. We ascribe this to ischaemia-induced mobilisation in these patients. Confirming our previous results, the 4-week de-novo treatment of atorvastatin in stable CAD evoked an increase only of CD45^dim^CD34^+^KDR^+^ EPC, confirming our previous study [24]. We thereby confirm that indeed only the fraction of CD45^dim^ cells harbours the “true” circulating EPCs [14].

In conclusion, the protocol adapted from the standardised ISHAGE protocol demonstrated a higher accuracy in detecting subsets of EPCs. This valid, rapid and easy to use quantification of EPC in clinical studies may be suitable to overcome interlaboratory discrepancies. Stratification with respect to the expression of CD45 indicates that specifically the CD45^dim^CD34^+^KDR^+^ cell population correlates with cardiovascular disease and is significantly regulated by statin treatment, suggesting that the CD45^dim^ fraction contains the prognostic relevant EPC populations. Therefore, future studies should prospectively address, whether the detection of CD45^dim^CD34^+^KDR^+^ cells is only useful as biomarker or whether this population of circulating cells indeed comprise cells with a high capacity for vascular repair.
